# Sensitivity study for ICON tidal analysis

**DOI:** 10.1186/s40645-020-00330-6

**Published:** 2020-05-22

**Authors:** Chihoko Y. Cullens, Thomas J. Immel, Colin C. Triplett, Yen-Jung Wu, Scott L. England, Jeffrey M. Forbes, Guiping Liu

**Affiliations:** 1 Space Sciences Laboratory, University of California Berkeley, Berkeley, USA; 2 Virginia Polytechnic Institute and State University, Blacksburg, USA; 3 University of Colorado at Boulder, Boulder, USA

**Keywords:** Atmospheric tides, ICON

## Abstract

Retrieval of the properties of the middle and upper atmosphere can be performed using several different interferometric and photometric methods. The emission-shape and Doppler shift of both atomic and molecular emissions can be observed from the ground and space to provide temperature and bulk velocity. These instantaneous measurements can be combined over successive times/locations along an orbit track, or successive universal/local times from a ground station to quantify the motion and temperature of the atmosphere needed to identify atmospheric tides. In this report, we explore how different combinations of space-based wind and temperature measurements affect the retrieval of atmospheric tides, a ubiquitous property of planetary atmospheres. We explore several scenarios informed by the use of a tidally forced atmospheric circulation model, an empirically based emissions reference, and a low-earth orbit satellite observation geometry based on the ICON mission design. This capability provides a necessary tool for design of an optimal mission concept for retrieval of atmospheric tides from ICON remote-sensing observations. Here it is used to investigate scenarios of limited data availability and the effects of rapid changes in the total wave spectrum on the retrieval of the correct tidal spectrum. An approach such as that described here could be used in the design of future missions, such as the NASA DYNAMIC mission (National Research Council, Solar and space physics: a science for a technological society, 2013).

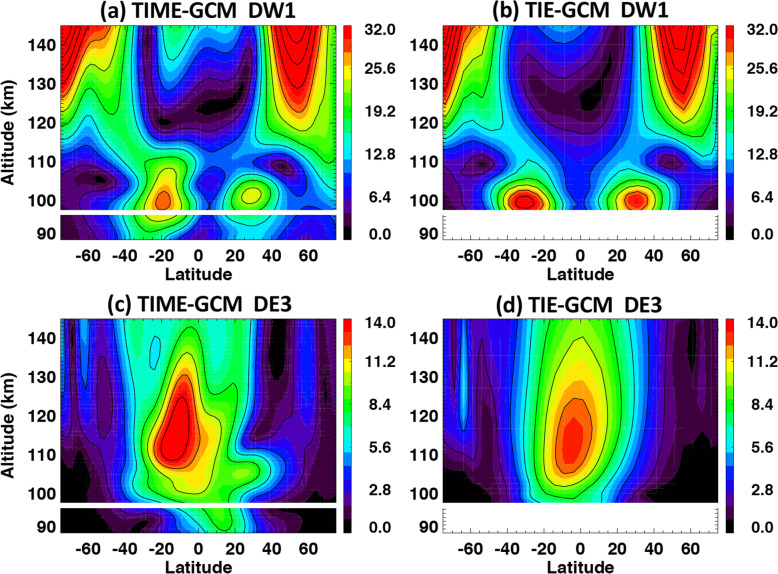

## Introduction

The ionosphere is affected by both solar and lower atmospheric inputs (Forbes et al. 2000). Atmospheric waves including planetary waves, tides, and gravity waves are the main agents for transporting energy and momentum from the lower atmosphere to the ionosphere and thermosphere. Among these waves, various observations and simulation studies have revealed that atmospheric tides originating in the troposphere and stratosphere have significant influences on the ionosphere (e.g., Immel et al., 2006; England et al., 2006; Forbes 2007; Forbes et al. 2018; England 2011; National Research Council 2013; and references therein). Because of their apparently dominant effects, it is critical to simulate realistic atmospheric tides in models. The upper atmospheric models (e.g., National Center for Atmospheric Research Thermosphere-Ionosphere-Electrodynamics General Circulation Model; NCAR TIE-GCM; Maute 2017) use various tidal forcing methods at their lower boundaries including the Global Scale Wave Model (GSWM; Hagan and Forbes 2002, 2003) and Climatological Tidal Model of the Thermosphere (CTMT; Oberheide et al. 2011). The GSWM is a model that solves for diurnal and semi-diurnal tides with realistic background conditions that originate in observations and/or empirical models. The CTMT estimates global tides by fitting Hough Mode Extensions (HMEs) to SABER (the Sounding of the Atmosphere using Broadband Emission Radiometry) and TIDI (the TIMED Doppler Interferometer) tidal determinations between ± 50° latitude and 80–105 km altitude from 2002 to 2008.

The Ionospheric Connection Explorer (ICON; Immel et al. 2018) mission (launched October 10, 2019) observes temperatures and winds from the mesosphere to the thermosphere continuously in day and night from 90 to 105 km in the latitude range of 10°S to 40°N, providing data adequate to evaluate the properties of a range of global tidal fields. The wind and temperature data will be fit with a set of HMEs that are representative of the tidal components present in the atmosphere (e.g., Forbes and Hagan 1982). Using these data, the TIE-GCM lower boundary will be specified using the ICON-HME tidal field in order to provide a realistic upper atmospheric and ionospheric simulation (Immel et al. 2018; Forbes et al. 2017; Maute 2017). The ICON-HME is a derived Level-4 mission data product that will be distributed as a product of the ICON mission (Forbes et al. 2017; Maute 2017).

Tidal analyses from satellite observations have several difficulties and limitations. The main issue is that a fit to tides by the HMEs demands an observational set with sufficient coverage in latitude, longitude, height, and local time to specify the important tidal components. To provide any data that is complete in this respect, observations are required to accumulate over certain periods of time. Given the interest in specifying the temporally variable tides over as short a period as possible, the performance of the HMEs with a partially full matrix is examined. A number of observational factors could reduce data availability, and so potentially further reduce the data available to the HME fit. These include the possibility of wind observations that have errors large enough to be flagged as “do not use,” short-term variations in the tidal field and short-term global-scale changes in the wind field originating in phenomena other than tides (e.g., planetary waves). It is important to understand the limitations and sensitivity of the tidal analysis and its influence on the obtained tidal field. In this study, using the ICON mission as an example, we will examine and discuss what can affect the tidal analysis and what needs to be considered when ICON is flown and when future missions are designed.

## Methods/Experimental

### HME (Hough Mode Extension)

**Fig. 1:**
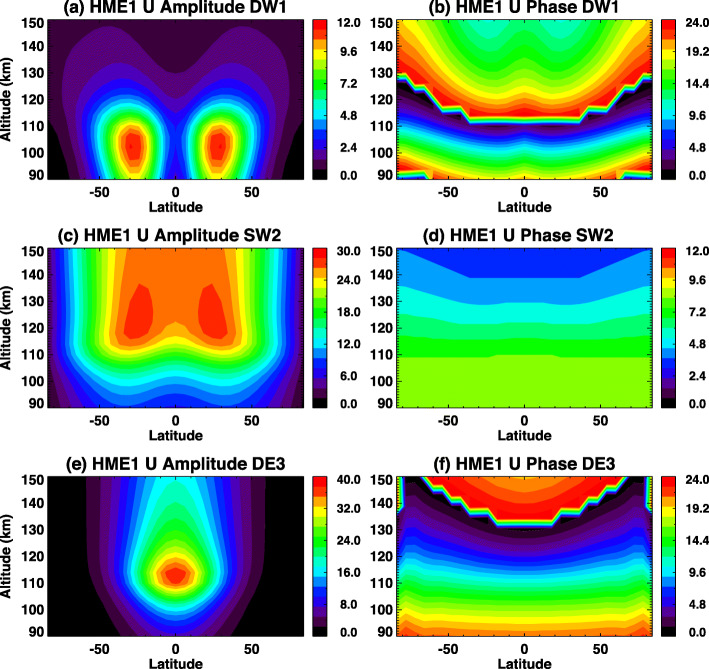
First symmetric HME amplitudes and phases for zonal wind (U) for **a**, **b** DW1; **c**, **d** SW2; and **e**, **f** DE3. HMEs are arbitrarily calibrated in amplitude to yield a maximum perturbation horizontal wind speed of 10 m/s (either zonal or meridional, whichever is larger) at 93 km. Absolute values of phases are also arbitrary

**Fig. 2:**
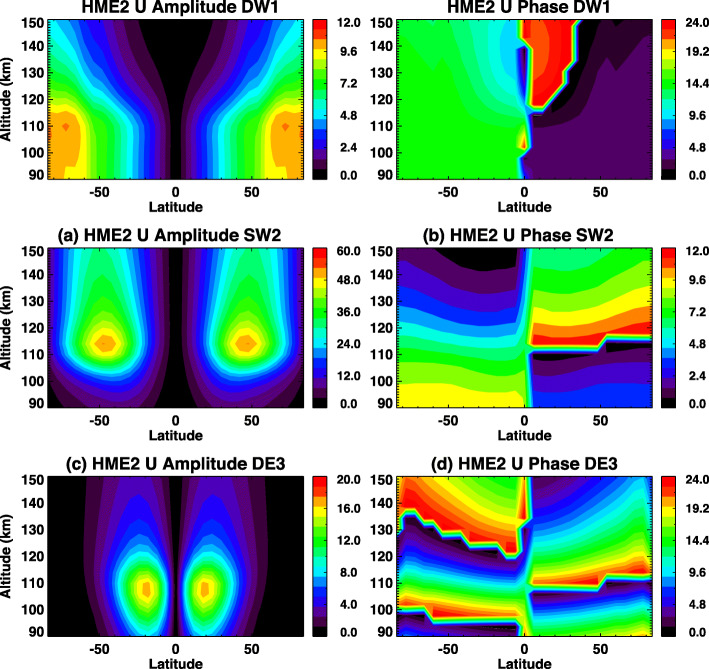
First antisymmetric HME amplitudes and phases for zonal wind (U) for **a**, **b** SW2 and **c**, **d** DE3. The latitude structure HME2 amplitude for DW1 does not resemble the first antisymmetric mode of classical tidal theory (maxima near ± 24° latitude) due to the strong influence of molecular dissipation, which couples the vertically-propagating DW1 with short vertical wavelength into an evanescent tidal mode

Hough’s original work in 1898 presented a critical mathematical representation of oscillations on a rotating earth as an eigenfunction-eigenvalue problem. The eigenfunctions are solutions to Laplace’s tidal equation and provide the latitudinal structures, whereas the eigenvalues are related to the depth of the oscillating fluid. The eigenvalues comprise an orthogonal set of functions that can be fit through a least-squares method to any appropriate dataset (Hough 1898). However, it is not physically meaningful to fit Hough functions to winds. To enable fits that include self-consistent relationships between perturbation fields in temperature, zonal, meridional and vertical wind, and density (T, u, v, w, ρ), the Hough Mode Extension (HME) was developed by Lindzen et al. (1977) and Forbes and Hagan (1982). The HMEs are computed using the GSWM with zero-velocity background winds and global mean vertical temperature and density profiles derived from the MSISE90 empirical model (Hedin 1991). Each HME maintains a self-consistent amplitude and phase relationship for the perturbation fields in temperature, zonal, meridional and vertical wind, and density (T, u, v, w, ρ). HMEs are arbitrarily calibrated in amplitude to yield a maximum perturbation horizontal wind speed of 10 m/s (either zonal or meridional, whichever is larger) at 93 km. Absolute values of phases are also arbitrary. HME fitting involves finding a single complex normalizing factor for the HMEs that determines absolute amplitudes and phases for all variables that best fit the input data in a least-squares sense. Figures 1 and 2 show structures of amplitude and phase in zonal wind tides for the DW1, SW2, and DE3 HMEs. These also show the first symmetric and first antisymmetric HMEs, respectively. For antisymmetric HMEs, antisymmetry enters through the phases.

In addition, the HMEs include the effects of dissipation, thus allowing extrapolation of the fitted fields into the thermosphere where the latitude and height dependences are no longer separable. Given tidal phases and amplitudes at a sufficiently diverse set of latitudes and altitudes, the HME provides a tool for fitting and reconstruction of the global atmospheric tidal field that is widely used. For example, Svoboda et al. (2005) tested and validated HME fitting against output from a general circulation model and applied the methodology to UARS (Upper Atmosphere Research Satellite) wind measurements. More recently, TIMED SABER and TIDI observations of temperatures and winds in the 80–105 km altitude range are used to create an HME-based tidal climatology of winds in the 80–400 km range and published as the CTMT dataset by Oberheide et al. (2011). Therefore, HME has been widely used and tested, and detailed HME methodologies are summarized in Forbes et al. (1994), and HME for ICON is summarized in Forbes et al. (2017) and references there in.

In this study, HMEs are fit to tidal temperatures and zonal winds from TIME-GCM data (see “TIME-GCM simulations” section). Unless otherwise specified, a latitude range of 10°S to 40°N and an altitude range of 90–105 km are used for the input data to the HME fit. The HME fit produces an output of tidal amplitudes and phases in zonal winds, meridional winds, vertical winds, temperatures, and densities/geopotential heights from 90°S to 90°N. In this study, our main focus is HME results at ~ 97 km, the height of TIE-GCM lower boundary and most results are shown at this altitude.

### ICON observations

Temperatures and winds will be measured by the Michelson Interferometer for Global High-Resolution Thermospheric Imaging (MIGHTI) instrument (Englert et al. 2017; Harlander et al. 2017; Harding et al. 2017). ICON-MIGHTI temperature and wind data need to be accumulated over time to adequately constrain the tides (in height, latitude, longitude, and local time) that form the basis for the HME fits (Immel et al. 2018). The ICON-MIGHTI observations are simulated using an orbital dynamics simulation of the observatory in science mode and a concurrent run of the TIME-GCM (see “TIME-GCM simulations” section). The wind and temperature observations come from locations approximately 1500 km from the observatory and a latitude range that is nominally from 10 to 15° north of the ICON orbit track, depending on the angle between observatory velocity vector and the local meridional plane.

**Fig. 3:**
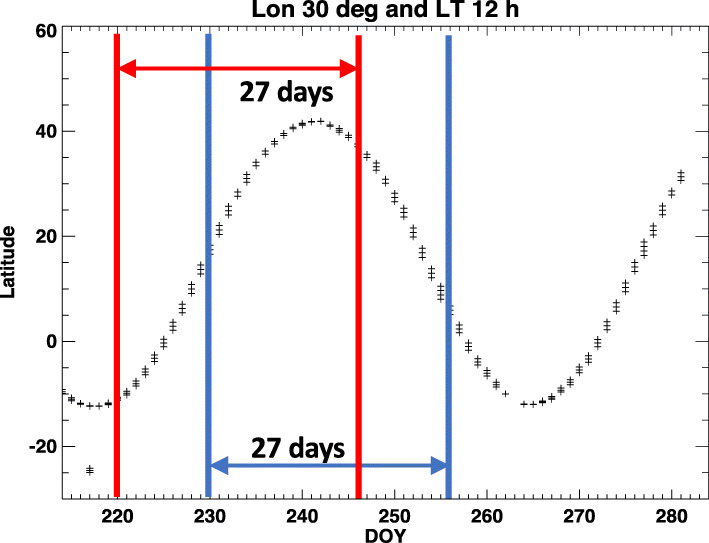
Latitudes of ICON sampling at 30° longitude and 12 local time within two 27-day analysis windows

**Fig. 4:**
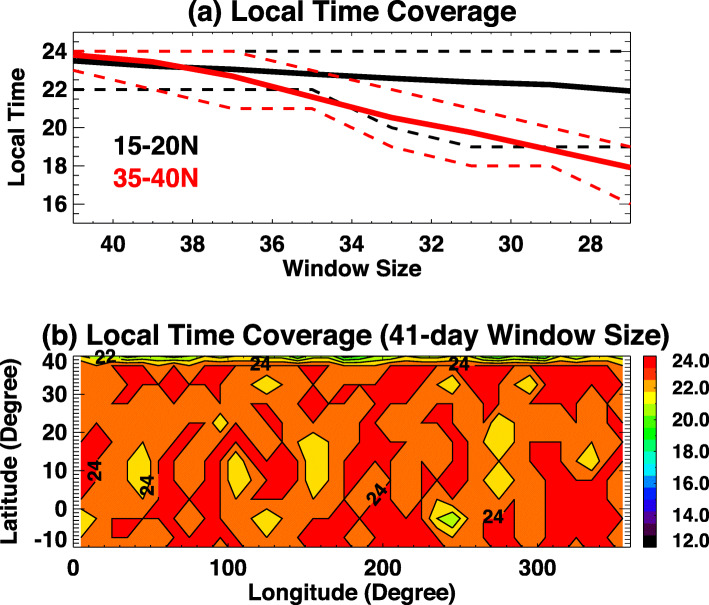
**a** Local time coverage in 2 latitude ranges. Solid lines indicate average local time coverage and dashed lines represents maximum and minimum local time coverage at each latitude range. **b** Contour plots of local time coverage with window size of 41 days, with 24 h indicating full local time coverage

ICON provides data with coverage that varies significantly if a window smaller than a full orbital precession cycle is used. Figure 3 shows the latitudinal coverage of ICON sampling at a longitude of 30°E and 12 local times (LT) within two 27-day analysis windows. In Fig. 3, ICON sampling from day of year (DOY) 220 to DOY 246 (27-day sampling) extends from 10°S to 40°N, the full range of possible latitudes. However, as the center of the analysis window advances from DOY 230 to DOY 256, the only latitudes measured are in the Northern Hemisphere. One can address this with a longer window, but the optimal window for revealing the shorter-period tidal variations uses the fewest days of data. Figure 4 shows local time coverage of ICON sampling vs. window size. A window size of *n* days indicates ICON data collection through day and night during *n* days to calculate tidal amplitudes and phases. Based on Fig. 4, a window size of 39 days provides 22 h of local time for the worst-case maximum excursion in latitude (with similar coverage in the south, not shown). Given our numerous experiments with different coverage, we can state that this local time coverage at every latitude provides wind and temperature data that is adequate to constrain the HME fit. As a baseline for the examination of missing data on the HME fits, full local time coverage with 41-day window will be used here.

### TIME-GCM simulations

Atmospheric simulations from TIME-GCM are used to conduct this sensitivity study (Roble and Ridley 1994). In order to have a realistic simulation for testing, the lower boundary at 10 hPa is specified by 3-hourly Modern-Era Retrospective analysis for Research and Applications (MERRA) data. The horizontal resolution is 2.5° × 2.5° (latitude × longitude), and the vertical resolution is four grid points per scale height. MERRA data are interpolated to TIME-GCM longitude-latitude grid. TIME-GCM uses gravity wave parametrization scheme based on Lindzen (1981). Comparison of tidal amplitudes from SABER observations and MERRA-forced TIME-GCM simulations can be found in Forbes et al. (2017). These TIME-GCM simulations conducted for the solar minimum year of 2009. The 3-hourly KP index and daily solar fluxes are used as input to the model. The model domain is from ~ 30 km (10 hPa) to ~500 km with 97 vertical levels. TIME-GCM data from DOY 220 to 260 (41 days window) are sampled based on the predicted ICON sampling locations for most of the sensitivity study. Detailed model setup is described in Häusler et al. (2014, 2015).

### TIE-GCM simulations

The NCAR TIE-GCM V2.0 is also used for this work (Maute 2017). In the course of the ICON mission, this will be the model that ingests the HME fit at its lower boundary of ~ 97 km altitude. Its performance vs the TIME-GCM runs in simulating the tidal spectrum is evaluated here. Zonal-mean wind, temperature, and geopotential height from Mass Spectrometer Incoherent Scatter Rader Extended (MSIS E00) (Picone et al. 2002) and the horizontal wind model (HWM07) (Drob et al., 2008) are specified at the lower boundary. In accordance with the above stated goal of comparing the TIME-GCM to the TIE-GCM forced by simulations of MIGHTI measurements of that same TIME-GCM atmosphere, diurnal and semi-diurnal tides are specified using HMEs developed in this study as described above. Detailed model setup and ionospheric simulations for the ICON mission can be found in Maute (2017). HME fits are performed on TIME-GCM output with ICON sampling for this study, and results are shown in the “Results and discussion” section.

## Results and discussion

### Sensitivity study

#### Sampling window size

**Fig. 5:**
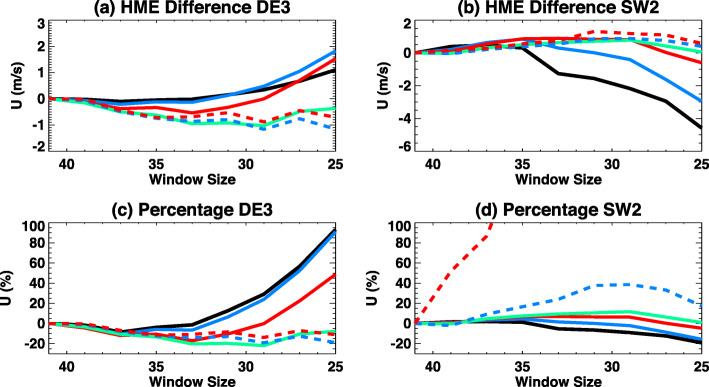
Comparisons of tidal amplitudes using different window size from 41 to 25 days. Colors represent different latitudes for solid-black (35°N–40°N), solid-blue (25°N–35°N), solid-green (15°N–25°N), solid-red (5°N–15°N), dashed-blue (5°S–5°N), and dashed-red (10°S–5°S). Amplitude differences between with and without ICON sampling are shown in **a** and **b** for DE3 and SW2, respectively. Percentages of these differences are shown in **c** and **d**

The effect on the HME fit of reducing the window size to less than 41 days is shown in Fig. 5. HME-determined tidal amplitudes using ICON sampling windows ranging from 41 down to 25 days are compared to tides determined from complete sampling. The latter are simply the actual averaged tidal amplitudes over the corresponding window size. Hence, differences of tides in Fig. 5 are simply representing the influence of varying ICON sampling on tidal extraction. DE3 (eastward-propagating diurnal tide with zonal wavenumber 3) and SW2 (westward-propagating semi-diurnal tide with zonal wavenumber 2) are selected to highlight due to the likely importance of each in influencing the ionosphere and also to provide diurnal and semi-diurnal tidal results. In this study, to represent different tidal components, the notation DWs or DEs will be used to indicate a westward or eastward-propagating diurnal tide, respectively, with zonal wavenumbers. “D” will be replaced with “S” for semi-diurnal tides. For example, DE3 will be eastward propagating diurnal tides with zonal wavenumber 3.

As expected, differences get larger as window size gets smaller because of missing local time coverage. However, largest absolute amplitude differences for DE3 and SW2 are ~ 2 m/s and ~ 3 m/s, respectively, within a 27-day window size from Fig. 5 a and b. These changes are ~ 20% as shown in Fig. 5 c and d. Blue and black lines in Fig. 5c and dashed-red and dashed-blue lines in Fig. 5d show significantly large changes in percentages, and these are due to very small tidal amplitudes in these latitude ranges. Absolute differences for these abnormal percentages are still less than ~ 2 m/s, indicating that only moderate influences are anticipated on the HME fits to these data. For temperature and meridional wind tides (not shown), the largest absolute amplitude differences are also ~ 2.5–3 m/s and ~ 1 K for DE3, respectively. Percentage changes of temperature and meridional wind tides for DE3 and SW2 are similar to zonal wind change shown in Fig. 5.

#### Terminator and missing local time

One potential problem of ICON observations is the possible gap in local time coverage near Earth’s solar terminator. Because of the rapid variation in the airglow profiles with altitude, the wind retrievals may have large uncertainties. Therefore, the effect of the absence of data at the terminator is examined in this section. DE3 results are mainly presented in this paper because of its dominant presence in the lower thermosphere during certain months. SW2 results are also shown here as an example of semi-diurnal tides.

**Fig. 6:**
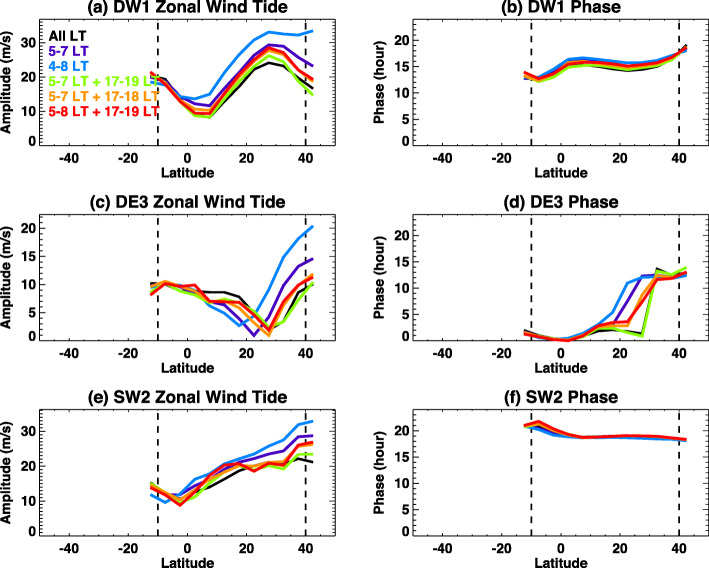
Line plots of **a**, **b** DW1; **c**, **d** DE3; and **e**, **f** SW2 tidal (left) amplitudes and (right) phases for zonal wind tides at 97 km. Different colors indicate different missing local times. For example, “5–7 LT + 17–19 LT” means that tidal amplitudes are calculated using data with missing local times of 5, 6, 7, 17, 18, and 19 LT

The amplitudes of the DW1, DE3, and SW2 tides retrieved from the tidal fits where data are excluded in specific local time ranges are shown vs latitude in Fig. 6. These tides are calculated from ICON-sampled TIME-GCM with MERRA lower boundary simulated for DOY 220 to 260, 2009. Tests using different gaps in local time are indicated by different colors, noted in the figure legend. Black represents the base case with full local time coverage. Blue lines, for example, indicate missing morning local times from 4 LT to 8 LT. For DW1, DE3, and SW2 (Fig. 6a, c, and e), the most notable features are that a 4–8 LT data gap results in the largest differences from the base case, with departures up to ~ 10–15 m/s in winds. On the other hand, cases of missing local time both in the morning and evening sector (green lines) (“5–7 LT + 17–19 LT”) show only changes of ~2–5 m/s in zonal winds. For DE3, the largest changes are where amplitudes of DE3 are normally small (less than 5 m/s). DE3 amplitudes between 10°S and 20°N where the DE3 peak usually lies show no significant influences from missing local time around the terminator. For DW1 and SW2, all missing data tests introduce only small changes in the observed phases of DW1 and SW2. The general latitudinal structures of DW1 and SW2 amplitudes are similar with and without missing local times, and missing local times from 5–7 LT (3 h) cause ~ 5 m/s changes, which translate to ~ 35% differences in SW2 and ~ 25% differences in DW1.

**Fig. 7:**
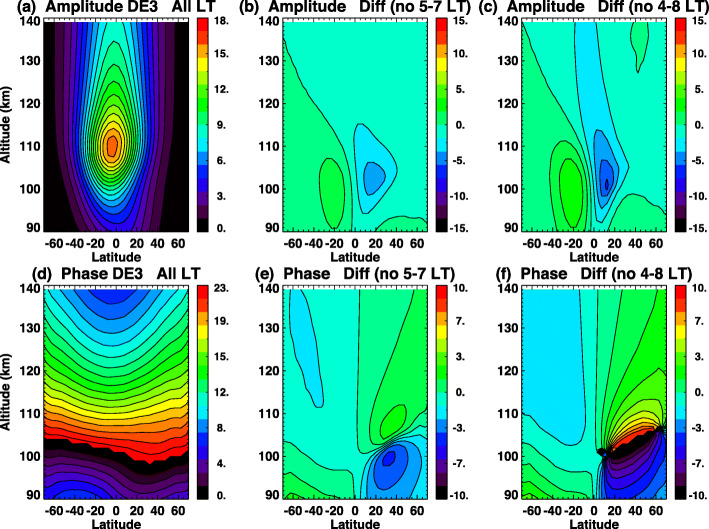
Height vs. latitude structures of DE3 HME **a**, **b**, **c** amplitudes and **d**, **e**, **f** phases. Left plots **a**, **d** show zonal wind tidal amplitudes (m/s) based on fits including all local times. Middle and right plots show differences of zonal wind tidal amplitudes between fits to data with complete all local time coverage and data with missing local time **b** and **e** from 5 LT to 7 LT and **c** and **f** from 4 LT to 8 LT

Effects of data gaps on the DE3 amplitudes and phases vs altitude and latitude are shown in Fig. 7. Here we use HME extensions above 97 km for these numerical experiments, although when applied in the ICON mission the extensions will be performed using the TIE-GCM. Figure 7 a and d illustrate amplitudes and phases of zonal wind based on the HME fit at 97 km wherein data from all local times are included. Figure 7 b and e illustrate differences in amplitude and phase from those in Fig. 7 a and d when observations in the 5–7 h LT range are absent. Differences in DE3 are greatest (2–4 m/s) around 110 km and in the 0–20°N range. Peak amplitudes of DE3 in Fig. 7a in this area are ~ 12–16 m/s, indicating that changes up to ~ 15–25% with 3 h of continuous missing data can occur. Figure 7 c and f show the same as Fig. 7 b and e but with 5 h of continuous missing data from 4 LT to 8 LT. The largest differences grow to ~ 30% and are again in the latitude range of 0–20°N in the 100–110 km altitude range. Changes in phases are greatest (2–4 h) in the latitude range of 20–40°N and altitude range of ~ 95–110 km with 3 h of missing local time. Small differences in amplitudes are approximately associated with the largest shifts of phases of DE3 in the Northern Hemisphere where, as noted in Fig. 6, the largest amplitude changes are found. From these results, we find that DE3 will be reasonably estimated even with terminator influences on local time coverage as long as missing local times are less than ~ 4 h continuously, and missing both dawn and dusk terminator data does not have large effects on DE3 tidal amplitudes.

**Fig. 8:**
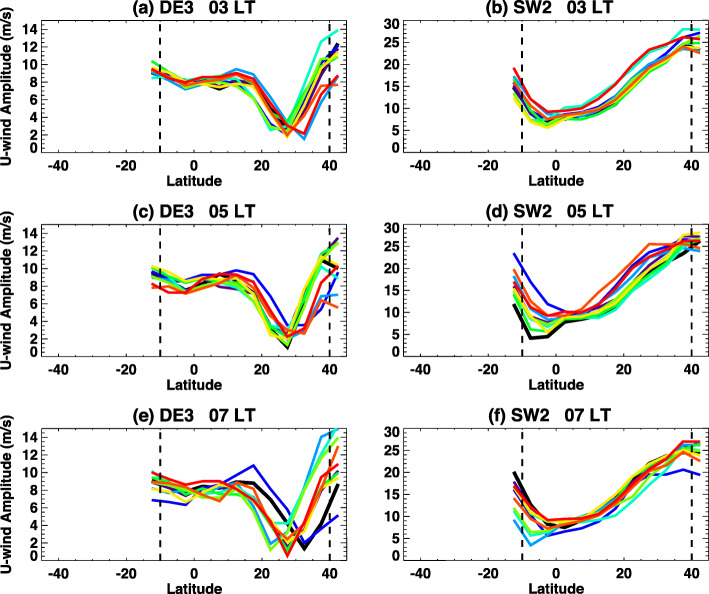
Line plots show **a**, **c**, **e** DE3 and **b**, **d**, **f** SW2 tidal amplitudes in zonal winds calculated using ICON-sampled TIME-GCM simulations at 97 km. Tidal amplitudes calculated using data with missing **a**, **b** 3 h; **c**, **d** 5 h; and **e**, **f** 7 h of local time randomly. Each panel contains 10 tidal amplitudes calculated using randomly generated local time gap

In addition to missing local times, effects of randomly missing data on the tidal analysis for DW1, DE3, and SW2 are examined here using an ensemble approach that effectively simulates a reduction in duty cycle of the instrument. Tides are estimated from observations limited to a total of 21, 19, and 17 h of local time coverage out of 24 h. Missing local times, in 1 h increments are randomly selected for these cases, with 10 separate, random-sampling cases fitted for each. The results are shown with 10 colors in Fig. 8. Tidal analysis with missing 3 h, 5 h, and 7 h LTs are shown. DE3 in Fig. 8 a, c, and e show the growing range of tidal amplitudes resulting from increasingly sparse data. SW2 fits in Fig. 8 b, d, and f show variations of similar percentage, but also possibly a more resilient fit to missing data, with the 7 h total missing data case no worse than the 3 h case.

#### Limited altitude coverage

**Fig. 9:**
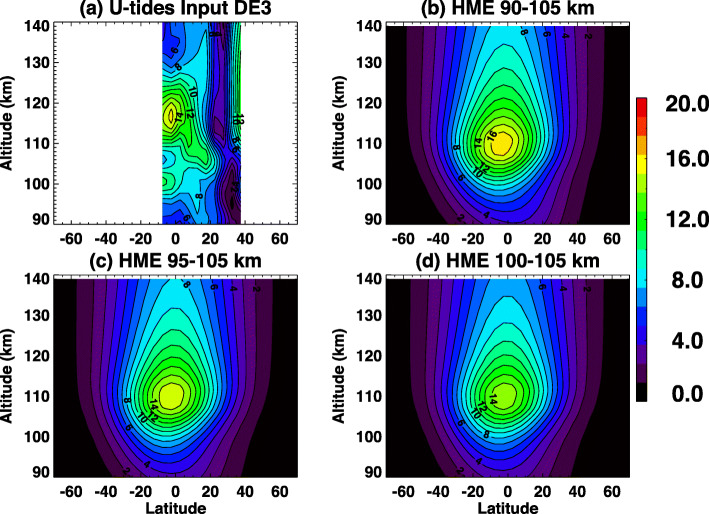
**a** ICON-sampled TIME-GCM DE3 zonal wind amplitudes (m/s) and remaining plots are HME DE3 zonal wind amplitudes based on fits to ICON-sampled TIME-GCM DE3 tidal amplitudes in the altitude range of **b** 90–105 km, **c** 95–105 km, **d** 100–105 km. White lines indicate 105 km to indicate ICON sampling altitude

All satellite observations have limited altitude coverage. For ICON, a mission requirement is to provide continuous observations of winds and temperatures from ~ 90 to ~105 km. This section tests the sensitivity of HME fits to both wider and more limited observational ranges. All analyses here use the latitude range of 10°S–40°N with full local time coverage for the tidal fit. Figure 9 a shows ICON-sampled TIME-GCM DE3 zonal wind amplitudes between 10°S and 40°N and 90–140 km; values between 90 and 105 km are the source data for the various HME fits shown in Fig. 9b–d. Figure 9b illustrates the height-latitude HME amplitude structure based on fitting to the baseline 90–105 km observations, and its vertical extension to 140 km. Additionally, DE3 zonal wind amplitudes determined via HME fits to 95–105 km, and more limited 100-105 km input altitudes are shown in Fig. 9c and d, respectively.

There are several points to be made concerning this figure, which elucidates several important points about the HME methodology and its application within the ICON mission. The first, which gets at the main purpose of this subsection, is that HME fitting of the input DE3 tide is very similar between the three cases, albeit with slightly different amplitudes. The amplitudes with inputs from 90 to 105 km are larger and closer to the actual DE3 amplitudes than those with 95–105 km and 100–105 km. Amplitudes from 100 to 105 km inputs are slightly smaller than the input tides. Although there are some differences, the conclusion is that HME fitting is not very dependent on complete altitude coverage of ICON data between 90 and 105 km.

A second point concerns the differences in structure between the input DE3 tide between 90 and 105 km in Fig. 9a, and those depicted in Fig. 9b–d. For HME fitting of DE3, only the first symmetric and first antisymmetric modes are utilized. There are two reasons. The first is that the first 3 modes of DE3 have vertical wavelengths (*λ*_*z*_) of 56, 30, and 19 km based on classical tidal theory. Molecular dissipation of these 3 modes increase as 1/($\rho {\lambda}_z^2$) where *ρ* is the background density. The first mode can effectively penetrate into the thermosphere above 100 km, the second mode significantly less so, and the third mode is not at all effective. A tide with the vertical wavelength of 19 km would also not be at all effective in generating electric fields in the dynamo region, since this process involves height-integrated conductivity-weighted winds. In a fitting process, the purpose of including higher-order modes is to capture higher-order horizontal structure, but doing so is not beneficial when those modes do not contribute to vertical coupling. Our experience furthermore shows that fitting to the low-order HME components is much more stable and reliable than to the higher-order components. For all of these reasons, HME fitting with respect to DE3 is restricted to the first symmetric and first antisymmetric HMEs, recognizing that not capturing all of the fine structure between 90 and 105 km is not consequential to the problem at hand.

A third point concerns the differences in height-latitude structure of DE3 between Fig. 9a and the rest of the panels in Fig. 9 at altitudes above 105 km. The feature above 110 km near 40°N is not representative of DE3 in the full-sampled TIME-GCM output but is more likely attributable to aliasing into the ICON sampling by one or more other waves (e.g., SE3) in this highly dynamic environment presented by the MERRA-forced TIME-GCM (see, e.g., Forbes et al. 2017). It is a good example of why diligent oversight of the HME fitting process will be required during the ICON mission. In this case, the feature did not appreciably influence the HME fitting since it does not project onto the DE3 HME structures. The differences in structure equatorward of 20°N, in particular the higher altitude of the peak near 117 km versus 110 km in Fig. 9b–d, may also be due in part to sampling but may also reflect a deficiency of the GSWM at these altitudes, i.e., an overestimate of mechanical dissipation. However, this is inconsequential insofar as ICON is concerned, since it is the TIE-GCM that will specify the dynamics at these altitudes. The reader is referred to the comparison between TIME-GCM and TIE-GCM tidal structures in the “Comparisons between TIME-GCM and HME forced TIE-GCM” section for more insights.

#### Limited observation variables

**Fig. 10:**
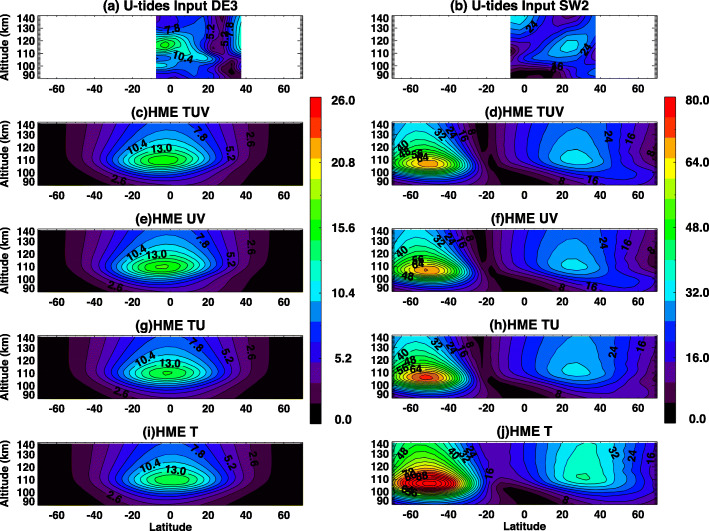
**a**, **b** ICON-sampled TIME-GCM DE3 and SW2 zonal wind tidal amplitudes that are used to fit HMEs (between 90–105 km), and **c**–**j** HMEs based on fits to **c**, **d** both temperature, zonal wind, and meridional wind; **e**, **f** zonal and meridional winds; **g**, **h** temperature and zonal wind; and **i**, **j** temperature only for (left) DE3 and (right) SW2

The ICON MIGHTI instruments measure temperature, zonal winds, and meridional winds. HME fits presented in prior subsections are performed using all three measurements. It is possible that ICON-MIGHTI, or any future mission, might measure only one or two variables and not all three. Hence, influences of missing variables on HME fit are examined here. Input tides and HME fits to data between 90 and 105 km with different input variables are shown in Fig. 10 for DE3 and SW2. For DE3 in Fig. 10, HME fits with TUV (temperature, zonal winds, and meridional winds), HME fits with UV (zonal wind and meridional wind), HME fits with TU (temperature and zonal wind), and HME fits with T (temperature) are very similar, and missing variables have small influences on DE3 in this case. As discussed in connection with Fig. 9, although HME fits are similar, there are some differences between input DE3 tidal amplitudes and HME DE3 amplitudes in particular above 35°N.

For SW2, input SW2 tides vs. HME fits with TUV, UV, and TU in 10°S–40°N compare well. HME fits with only T (temperature) generally overestimate tidal amplitudes in this case. Therefore, our results indicate that HME fits works better if two or more variables are used to constrain it. This is consistent with conclusions drawn by Svoboda et al. (2005).

### Comparisons between TIME-GCM and HME forced TIE-GCM

As the last part of this current work, the veracity of a TIE-GCM simulation with ICON-HME tidal forcing is demonstrated here. For the ICON mission, tidal fields obtained from HME-ICON will force the lower boundary of the TIE-GCM. To simulate a realistic case, ICON-sampled TIME-GCM data are used to calculate tidal amplitudes and phases between 90 and 105 km in the latitude range of 10°S–40°N. The HME fits to these tidal data are then used to force the TIE-GCM lower boundary at ~ 97 km. One reason to use ICON-HMEs to force the TIE-GCM is to generate realistic tidal fields in TIE-GCM above 97 km. A goal of this test is to see if tidal fields from TIME-GCM above 97 km and TIE-GCM above 97 km are similar. Only tidal amplitudes are shown in this section. Detailed analysis of ICON forced TIE-GCM are discussed in Maute (2017).

**Fig. 11:**
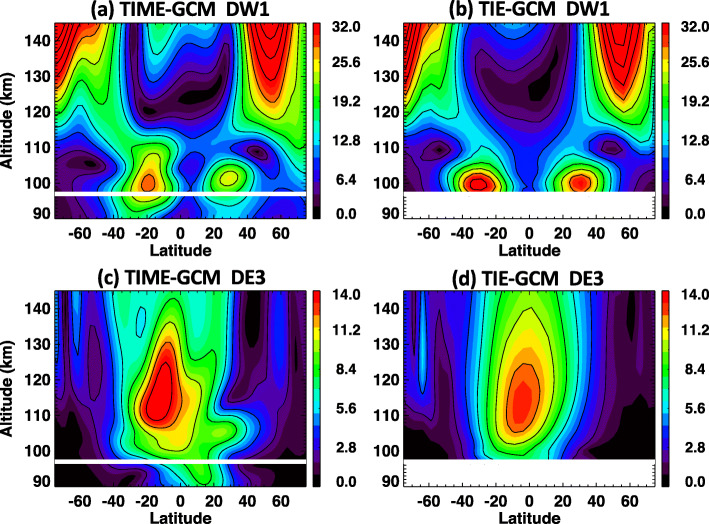
**a**, **c** TIME-GCM and **b**, **d** TIE-GCM simulations of **a**, **b** DW1 and **c**, **d** DE3. White lines indicate where the 97 km lower boundary of TIE-GCM is located and where the HME input is introduced

Figure 11 a shows DW1 tidal amplitudes obtained from the TIME-GCM simulation that covers the altitude range from 30 to ~ 500 km. A white line in Fig. 11a indicates the lower-boundary of the TIE-GCM. Using TIME-GCM tidal data in 10°S–40°N and 90–105 km, HME fits are performed. Then these HME tidal fields are used to force the TIE-GCM, and results are shown in Fig. 11b. In TIME-GCM for DW1, there are two large peaks in the latitude range of 60–80°S and 40–60°N from 120 to 150 km, and these two peaks can also be found in TIE-GCM. These are signatures of the diurnal tide excited in situ by absorption of solar EUV radiation. From 90 to ~ 110 km, there are also two peaks around 20°S and 30°N, which are captured by TIE-GCM as well, and these are due to DW1 excited mainly in the troposphere. These DW1 amplitudes are reduced in TIME-GCM compared to TIE-GCM, and this is possibly attributable to inclusion of a Lindzen-based gravity wave parameterization in the TIME-GCM (Lindzen 1981; Liu and Roble 2002) below 120 km, which is known to reduce DW1 amplitudes (Miyahara and Forbes 1991, 1994; Forbes et al. 1991).

Figure 11 c and d show comparisons between TIME-GCM and HME-forced TIE-GCM for DE3. General structures of DE3 are well captured by TIE-GCM, though there are differences between TIME-GCM and TIE-GCM above 110 km. One of the causes of differences between TIME-GCM and TIE-GCM results can be inclusion of a gravity wave parametrization scheme in the former, which may also introduce mean wind effects that produce some distortions. Another is the presence of higher-order modes of DE3 in the TIME-GCM which are damped out by molecular dissipation above 110 km, as explained in the “Limited altitude coverage” section. Our overall conclusion is that lower boundary conditions extending pole to pole based on HME fitting to ICON observations that are restricted to 12°S to 40°N and 90–105 km altitude can lead to realistic tidal structures in the TIE-GCM. This capability will enable us to significantly advance ICON science within an observation- and physics-based modeling framework.

## Conclusions

A sensitivity study of tidal analyses pertaining to potential satellite observational sampling constraints are discussed in this paper, including missing local times, limited altitude coverage, and missing observed variables. The most critical of these is that any systematic gap in data in some local time range (possibly the terminators) has the potential to cause a 20–30% of change in the amplitudes of HMEs fitted to tides retrieved from observations with these gaps, though this effect depends on the tide and whether the gaps are symmetric about noon (as is the case with the terminators). Comparatively, an under-sampled parameter space in local time, if randomly distributed, has a more predictable effect of introducing a slowly increasing overall error in the HME that is roughly evenly distributed in latitude. In any case, the effect of missing local times needs careful consideration when analyzing tides from satellite observations. Further, we show that a reduction in the altitude range of observations in the MLT region does not adversely affect the retrieval of tidal components by the HME fitting procedures.

Tidal amplitudes and phases between TIME-GCM and TIE-GCM simulations above 97 km are compared. TIE-GCM simulations are conducted using HME tidal lower boundary from TIME-GCM 90-105 km tidal amplitudes and phases. Results confirm that TIE-GCM with HME tidal field as a lower boundary input can approximate well the DE3 and DW1 TIME-GCM tidal fields in the mesosphere and the thermosphere. Thus, the HME-forced TIE-GCM provides a representation of the MLT that is similar to a fully simulated model with inputs originating in the middle atmosphere. As tides are important for reproducing ionospheric variability, further study of potential influences of missing-local time on the ionospheric simulations will be conducted.
